# Clinical significance of the series of CYP2C9*non3 variants, an unignorable predictor of warfarin sensitivity in Chinese population

**DOI:** 10.3389/fcvm.2022.1052521

**Published:** 2022-11-24

**Authors:** Dongxu Wang, Hualan Wu, Min Dong, Qing Zhang, Anxu Zhao, Xinlong Zhao, Jia Chong, Minghui Du, Yan Wang, Haifeng Shi, Shuanghu Wang, Fang Wang, Jianping Cai, Jiefu Yang, Dapeng Dai, Hao Chen

**Affiliations:** ^1^Department of Cardiology, National Center of Gerontology, Beijing Hospital, Beijing, China; ^2^Arrhythmia Center, National Center for Cardiovascular Diseases, Fuwai Hospital, Chinese Academy of Medical Sciences, Beijing, China; ^3^Laboratory of Clinical Pharmacy, The People’s Hospital of Lishui, The Sixth Affiliated Hospital of Wenzhou Medical University, Lishui, China; ^4^The Key Laboratory of Geriatrics, National Centre of Gerontology, Beijing Hospital, Beijing Institute of Geriatrics, Beijing, China

**Keywords:** warfarin, *CYP2C9*, gene polymorphism, allele, anticoagulation

## Abstract

**Backgrounds:**

Gene polymorphisms are critical for variations in warfarin dose. To date, more than 70 *CYP2C9* alleles have been identified. This study was designed to clarify the clinical significance of CYP2C9*non-3 variants to warfarin sensitivity in Chinese Han patients.

**Methods:**

The entire *CYP2C9* gene region was sequenced in 1,993 individuals, and clinical data and *VKORC1* genotypes were collected from 986 patients with atrial fibrillation treated with warfarin. The SKAT-O method was used to analyze the effects of CYP2C9*non-3 variants on warfarin sensitivity.

**Results:**

A total of 20 *CYP2C9* variants were identified, of which four were novel. Carriers with CYP2C9*non-3 variants may have lower warfarin dose requirements, and similar to CYP2C9*3, CYP2C9*non-3 variants are clearly relevant to warfarin-sensitive and highly sensitive responders.

**Conclusion:**

Our results showed that, besides CYP2C9*3, the series of CYP2C9*non-3 variants is an unignorable predictor for warfarin sensitivity in Chinese population. From a safety consideration, people carried such variants may need a preferred choice of NOACs when they started anticoagulation therapy.

## Introduction

Despite the increasing clinical applications of novel oral anticoagulants (NOACs), warfarin remains the first-line choice for prophylaxis and treatment of thromboembolic events in various diseases, especially in resource-limited regions and in patients with specific indications (1). However, the clinical application of warfarin is limited by its narrow therapeutic window and large interindividual differences. Anticoagulation is affected by various genetic and clinical factors, and frequent blood sampling is required to monitor the international normalized ratio (INR) during warfarin treatment (2, 3). Warfarin is an important cause of hospital admission due to adverse drug events (4).

Genetic factors, including *CYP2C9, VKORC1*, and *CYP4F2*, play important roles in warfarin dosing variations (5). Studies have revealed that patients with different *CYP2C9* and *VKORC1* genotypes have varying warfarin sensitivity (6, 7). Carriers of the *VKORC1-1639G* allele have higher warfarin dose requirements, and CYP2C9*2 and *3 variants are associated with increased warfarin sensitivity. Furthermore, the ENGAGE-TIMI 48 study has shown that the advantage of NOACs over warfarin in reducing the early bleeding risk mainly in subgroup patients who were sensitive or highly sensitive to warfarin (6).

The gene polymorphisms of *CYP2C9* are the most abundant. To date, over 70 *CYP2C9* alleles have been identified, and their distributions in different populations differ significantly (8). CYP2C9*2 and *3 have been commonly investigated as allele variants in previous studies. Although CYP2C9*3 is the most common variant in the Chinese Han population, it occurs less frequently than in European and Latino populations, whereas CYP2C9*2 is extremely rare (9). Sequencing these two variants alone may fail to effectively identify patients sensitive to warfarin.

Several studies by Dai et al. found nearly 40 *CYP2C9* allele variants in Han Chinese (10, 11), and all variants except CYP2C9*3 are rare in the Chinese Han population. The total CYP2C9*non-3 allele frequency is 2.58%. *In vitro* and *in vivo* studies have demonstrated that most of these are associated with lower drug metabolic activities (12–14). This indicates that CYP2C9*non-3 variants are not uncommon in the Chinese Han population, and similar to *3, carriers with these variants may have lower warfarin dose requirements. The Clinical Pharmacogenetics Implementation Consortium (CPIC) guidelines published in 2017 also suggested that the effects of rare variants CYP2C9*5, *6, *8, and *11 should be considered in individuals of African descent (15). However, the effects of various CYP2C9*non-3 alleles on stable warfarin doses in Han Chinese have not been fully studied. CYP2C9*non-3 variants may indicate increased sensitivity to warfarin in patients. From a safety consideration, people with such variants may need a preferred choice of NOACs when they started anticoagulation therapy.

## Materials and methods

### Study participants

From January 2013 to October 2019, 1 993 Chinese Han patients with atrial fibrillation were recruited at Beijing Hospital, China. From them, 986 subjects were treated with warfarin as anticoagulant therapy and 1 007 with NOACs. Inclusion criteria for patients with atrial fibrillation were as follows: aged ≥ 18 years, met the indications for anticoagulant therapy, and agreed to receive anticoagulant therapy for over 3 months. Exclusion criteria were as follows: blood pressure ≥ 170/110 mmHg, complications with active bleeding, abnormal coagulation function, malignant tumor, and pregnancy. The study protocol was approved by the Ethics Committee of the Beijing Hospital. All the participants provided written informed consent.

We sequenced the *CYP2C9* genotypes of all patients and recorded the demographic and clinical information of 986 patients with atrial fibrillation treated with warfarin, including age, sex, height, weight, serum creatinine levels, smoking status, concomitant medication, and warfarin maintenance dose (mg/d). Warfarin maintenance dose was defined as the mean daily warfarin dose for patients with warfarin treatment ≥ 7 d after at least 2 consecutive (at least 7–14 d apart) INR within the target range (2.0–3.0), while the warfarin dose was not changed (16). At the same time, 986 patients were sequenced for the *VKORC1–1639G* > *A* genotype (*AA, GA*, and *GG*).

### Research methods

Peripheral venous blood (2.0 mL) was drawn from each subject using an EDTA anticoagulant. Genomic DNA was extracted from the leukocytes by precipitation. Primers used for amplification and sequencing are listed in Table 1 (TIANYI HUIYUAN BioTech, Beijing, China). Following our previously reported methods (10), all *CYP2C9* exons, exon-intron junction regions, and promoter regions were screened. PCR was carried out using the 25 μL reaction system, including 100 ng/μL genomic DNA, 1.5U LA Taq DNA polymerase (Takara Bio, Shiga, Japan), 1 × GC buffer I, 0.4 mM of dNTP, and 0.2 μM of each primer. The PCR cycling parameters were as follows: pre-denaturation at 94°C for 2 min, 35 cycles of denaturation at 95°C for 30 s, annealing at 60°C for 30 s, extension at 72°C for 30 s to 3 min, and final extension at 72°C for 5 min. The PCR products were verified by 0.8% agarose gel electrophoresis. The verified PCR products were digested with 4.8 U of shrimp alkaline phosphatase (Promega, WI, USA) and 1.5 U of exonuclease I (New England Biolabs, MA, USA). The DTCS Quick Start sequencing kit (Beckman-Coulter, CA, USA) was used to sequence the target DNA fragments using the dideoxychain-termination method, and the target DNA fragments were purified by ethanol precipitation. Purified sequencing products were sequenced using a CEQ 8000 DNA Sequence (Beckman-Coulter, CA, USA), and the sequencing results were analyzed using LaserGene software (DNASTAR, Madison, WI, USA) and Chromas software (Technelysium, South Brisbane, Australia). All sequencing results were manually verified by at least two researchers to ensure accuracy. Table 1 shows the novel *CYP2C9* allele variants detected in this study.

**TABLE 1:** Primers used for amplification and sequencing of *CYP2C9* gene.

	Primer sequence	Length of amplification (bp)
Exon 1	F: GACAATGGAACGAAGGAGAACAAGACCAAAGGAC R: GGTTTCATTCCACTATTTCTGACACTGACA	548
Exon 2 and Exon 3	F: TACAAATACAATGAAAATATCATG R: CTAACAACCAGGACTCATAAT	690
Exon 4 and Exon 5	F: CTATTCTTGCCCTTTCCATCTCAGTGCCCAG R: CTTGTTATTGGTCTATTCAGGGATTTGACT	2,261
Exon 6	F: TAGGCAAGCATGGAATAAGGGRGTAGG R: AATCACCRTTAGTTTGAAACAGATTACAGC	497
Exon 7	F: CCCCTGAATTGCTACAACAAA R: ACCCGGTGATGGTAGAGGTT	345
Exon 8 and Exon 9	F: CTTCTTTGGAACGGGATTTCCTCATCTGC R: TCTGTCCTTATCATTTTGAGAACCAGCAT	3,583

F, forward; R, reverse; bp, base pair.

### Statistical methods

SPSS 25.0 software and R software were used for statistical analysis, and statistical significance was defined as *p* < 0.05. The allele and genotype frequencies were calculated using the direct counting method, and the Hardy–Weinberg genetic equilibrium law was analyzed using the χ^2^-test. Count data are expressed as percentages (%). The measurement data are expressed as mean and standard deviation (x ± s). The main parameters were tested for normality using the Shapiro–Wilk test. Differences between different genotype groups were tested using the χ^2^-test and analysis of variance. An independent sample *t*-test was used to compare the mean warfarin doses between the groups. The χ^2^-test was used to compare the proportion of warfarin-sensitive and highly sensitive patients between groups. Rare variant association analysis (SKAT-O) was used to analyze the effects of CYP2C9*3 and *non-3 variants on warfarin sensitivity using the “SKAT” R package.

## Results

CYP2C9*3 was the most common variant, accounting for 5.03% of the total variants. All variants, except CYP2C9*3, were rare in the Han population. Nineteen CYP2C9*non-3 variants were identified in this study (Table 2), including 15 previously reported variants (CYP2C9*2, *13, *16, *27, *29, *31, *33, *39, *42, *46, *50, *58, *59, *60, and *62) and four new non-synonymous variants. They have been designated as CYP2C9*72–*75 by the Pharmacogene Variation Consortium (Supplementary Figure 1). The frequencies of various rare *CYP2C9* variants ranged from 0.03 to 0.18%. The total frequency of all CYP2C9*non-3 variants was 1.20%. CYP2C9*13 was the most common *non-3 variant, with an allele frequency of 0.18%.

**TABLE 2:** Allele distribution of *CYP2C9* in Han Chinese.

Allele	Region	Haplotype	*N* = 3,986	Frequency (%)
*2	Exon 3	−1188T > C, −1096A > G, −620G > T, −485T > A, −484C > A, 3608C > T (R144C)	5	0.13
*3	Exon 7	−1911T > C, −1885C > G, −1537G > A, −981G > A, 42614A > C (I359L), 50298A > T	200	5.02
*13	Exon 2	3276T > C (L90P)	7	0.18
*16	Exon 6	33497A > G (T299A)	3	0.08
*27	Exon 3	3627G > T (R150L)	2	0.05
*29	Exon 6	33437C > A (P279T)	6	0.15
*31	Exon 7	42519 (I327T)	1	0.03
*33	Exon 3	3573G > A (R132Q)	3	0.08
*39	Exon 2	3300G > T (G98V)	1	0.03
*42	Exon 3	3549 G > A (R124Q)	2	0.05
*46	Exon 3	3623G > A (A149T)	2	0.05
*50	Exon 5	10462C > T (P227S)	1	0.03
*58	Exon 7	42548C > A (P337T)	1	0.03
*59	Exon 9	50173 (I434F)	1	0.03
*60	Exon 9	50273T > C (L467P)	4	0.10
*62	Exon 3	3551 (R125C)	1	0.03
*72 ^†^	Exon 3	3624C > T (A149V)	1	0.03
*73 ^†^	Exon 3	3626C > T (R150C)	1	0.03
*74 ^†^	Exon 4	8757G > T (Q214H)	1	0.03
*75 ^†^	Exon 8	47454A > C (N418T)	1	0.03

^†^ Novel variants detected in the study.

Among the 986 patients receiving warfarin anticoagulant therapy, 640 were male (64.9%), with a mean age of 68.1 (± 10.1) years. In terms of concomitant medications, 273 patients were prescribed statins and 203 were prescribed amiodarone. *AA* was the main *VKORC1* (*-1639G/A*) genotype, accounting for 75.1% of the total. *GA* and *GG* were detected in 222 (22.5%) and 24 (2.4%) cases, respectively. The subjects were divided into three groups according to the *CYP2C9* genotype: wild-type CYP2C9*1/*1, CYP2C9*3 variant carriers, and CYP2C9*non-3 carriers. Table 3 shows the baseline data for each group.

**TABLE 3:** Baseline characteristics of the study population.

Variables	CYP2C9*1/*1 (*n* = 875)	CYP2C9*3 carriers (*n* = 89)	Non*3 carriers (*n* = 22)	*P*-value
Age (x ± s, years)	68.0 ± 10.2	67.9 ± 9.0	70.7 ± 7.9	0.456
Height (x ± s, cm)	167.3 ± 8.1	165.7 ± 7.7	165.9 ± 7.6	0.146
Weight (x ± s, kg)	71.7 ± 13.2	69.9 ± 13.2	69.1 ± 11.4	0.328
Male gender, *N* (%)	565 (64.6%)	59 (66.3%)	7 (68.2%)	0.897
Smoking, *N* (%)	154 (17.6%)	15 (16.9%)	4 (18.2%)	0.982
SCr (x ± s, mmol/L)	76.1 ± 17.1	76.8 ± 22.2	75.2 ± 18.7	0.908
Hypertension, *N* (%)	320 (36.6%)	33 (37.1%)	6 (27.3%)	0.664
LVEF (x ± s,%)	61.1 ± 9.5	62.0 ± 8.2	58.9 ± 9.1	0.379
Takes statins, *N* (%)	244 (27.9%)	23 (25.8%)	8 (36.4%)	0.615
Takes amiodarone, *N* (%)	182 (20.8%)	16 (18.0%)	7 (31.8%)	0.358
*VKORC1-1639AA, N* (%)	650 (74.3%)	73 (82.0%)	17 (77.3%)	0.268
*VKORC1-1639GA, N* (%)	202 (23.1%)	15 (16.9%)	5 (22.7%)	0.408
*VKORC1-1639GG, N* (%)	23 (2.6%)	1 (1.1%)	0 (0%)	0.515

SCr, Serum creatinine; LVEF, left ventricular ejection fraction.

The warfarin dose requirements were obtained in 14 carriers with CYP2C9*non-3 variants, all of which were heterozygous (Table 4). CYP2C9*1/*13 was the most common variant. The mean doses in individuals with CYP2C9*1/*13 were 1.63 ± 0.28 mg/d, significantly lower than that of CYP2C9*1/*1 (*P* < 0.001).

**TABLE 4:** Stable warfarin doses in different *CYP2C9* genotypes carriers.

*CYP2C9* genotype	*N*	Warfarin dose (mg/d)
*1/*1	875	3.45 ± 1.46
Carriers with *3	89	2.15 ± 0.85
*1/*3	87	2.18 ± 0.83
*3/*3	2	0.63 ± 0.18
Carriers with *non-3	22	2.51 ± 1.74
*1/*2	1	2.50
*1/*13	5	1.63 ± 0.28
*1/*16	1	1.39
*1/*27	1	4.50
*1/*29	3	5.5 ± 0.71
*1/*31	1	1.50
*1/*33	1	0.75
*1/*39	1	1.50
*1/*42	1	4.50
*1/*50	1	5.25
*1/*58	1	1.00
*1/*59	1	1.50
*1/*60	2	1.00 ± 0.35
*1/*62	1	2.50

**FIGURE 1:**
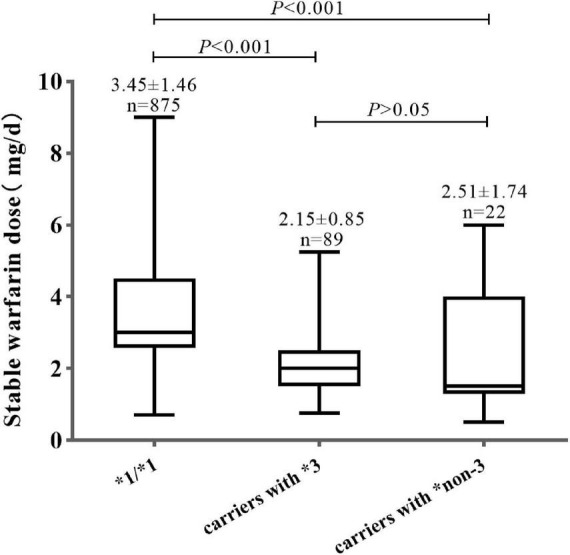
Warfarin dosage distribution in different *CYP2C9* genotype groups.

As shown in Figure 1, the mean warfarin dose of carriers with CYP2C9*non-3 was 2.51 ± 1.74 mg/d, which was significantly lower than that of wild-type carriers (*P* < 0.001). There were no significant differences in stable warfarin doses between carriers of CYP2C9*3 and *non-3 variants (*P* > 0.05).

The stable warfarin doses of the 986 patients ranged from 0.50 to 9.00 mg/d, with a median dose of 3.00 mg/d. Based on the warfarin dose distribution in the study population, warfarin sensitivity was classified into three categories (sensitive, highly sensitive, and others). Patients whose warfarin maintenance doses were within the lowest 20% dose (0.50–2.20 mg/d) were defined as warfarin-sensitive responders. The warfarin highly sensitive responders had the lowest 10% dose distribution (0. 50–1.35 mg/d).

**FIGURE 2:**
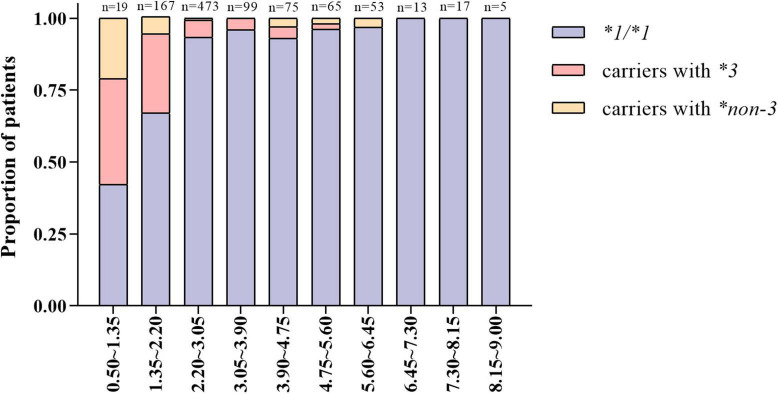
Distribution characteristics of carriers with different *CYP2C9* genotypes under varying warfarin dose groups.

The patients were divided into 10 groups according to the stable warfarin dose. As shown in Figure 2, patients with lower warfarin maintenance doses were more likely to carry the CYP2C9*3 and *non-3 variants. The proportion of carriers with CYP2C9*3 and *non-3 showed a tendency to increase gradually with decreasing warfarin maintenance doses. Among warfarin-sensitive and highly sensitive patients, the proportion of carriers with CYP2C9*3 was 28 and 37%, respectively, and the proportion of carriers with CYP2C9*non-3 was 7.0 and 21%, respectively. Additionally, a few carriers of CYP2C9*3 and *non-3 variants had increased warfarin dose requirements.

SKAT-O analysis showed that carrying CYP2C9*3 and *non-3 variants was significantly associated with warfarin sensitivity (*P* < 0.001) (Table 5). The impact persisted after adjustment for the *VKORCI-GA, GG*, and clinical variables. Similar to CYP2C9*3, CYP2C9*non-3 variants are significant for warfarin-sensitive and highly sensitive responders.

**TABLE 5:** Results of CYP2C9*3 and *non-3 variant association analysis (SKAT-O) with warfarin maintenance doses.

	*P-*value					
Responders	Model 1		Model 2		Model 3	
	*3	*non-3	*3	*non-3	*3	*non-3
Sensitive	7.91E-24	3.71E-06	2.72E-22	4.35E-06	8.29E-25	1.20E-06
Highly sensitive	0.0003	0.0002	0.0005	0.0002	0.0007	0.0002

Model 1, crude; Model 2, adjusted for *VKORC1-1639GA*, GG; Model 3, adjusted for *VKORC1-1639GA*, GG, age, sex, height, weight, smoking, SCr, concomitant use of amiodarone and statins.

**FIGURE 3:**
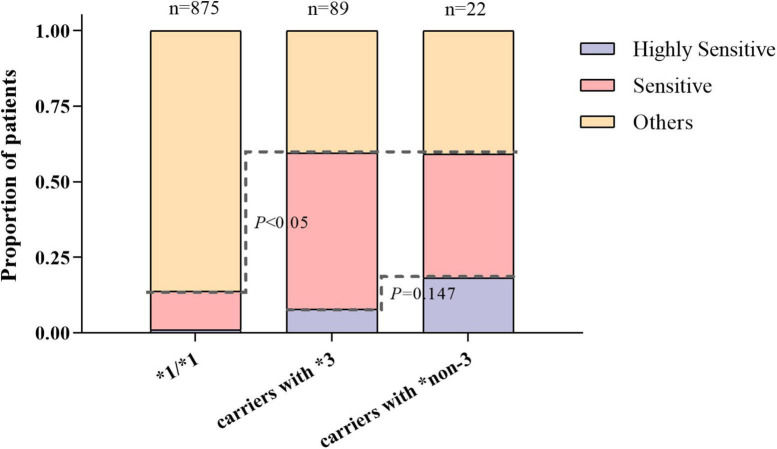
The distribution of warfarin sensitive and highly sensitive responders with different *CYP2C9* genotypes.

Figure 3 shows the distribution of warfarin-sensitive and highly sensitive responders with different *CYP2C9* genotypes. The proportion of warfarin-sensitive responders in carriers with CYP2C9*3 and *non-3 variants was comparable (60% vs. 59%), which was significantly higher than that of wild-type carriers (13%, *P* < 0.05).

Additionally, a higher proportion of carriers with CYP2C9*non-3 were warfarin highly sensitive responders compared to CYP2C9*3, but the difference was not significant (18% vs. 8%, *P* = 0.147). This suggests that both CYP2C9*3 and CYP2C9*non-3 variants had good predictive values for individual warfarin sensitivity. CYP2C9*non-3 variants had a higher predictive value for highly sensitive responders.

## Discussion

At present, over 70 *CYP2C9* alleles are known, and their distributions in different populations differ significantly (8). CYP2C9*2 and *3 are two commonly investigated *CYP2C9* allele *variants*. However, both alleles are more common in European populations, and relatively rare in Asian and African populations (8, 17). CYP2C9*3 is the most common variant in Han Chinese, with an allele frequency of 4%–5%, whereas CYP2C9*2 is extremely rare (9). Recently, the effects of *CYP2C9* alleles, other than CYP2C9*2 and *3 on stable warfarin doses, have received considerable attention. Particularly, CYP2C9*5, *6, *8, and *11 have been found to be important allele variants in people of African ancestry (18–20), and warfarin maintenance doses were significantly lower in carriers of these alleles. In contrast, there is less evidence on the effects of other rare *CYP2C9* variants on warfarin maintenance dose. These variants have been reported in only a few case reports, and further studies are still lacking (21–23).

In this study, 20 *CYP2C9* variants were identified by direct sequencing of all exons in the *CYP2C9* gene, including four new non-synonymous variants, which have been designated as CYP2C9*72–*75. Unlike racial and ethnic groups (8), all variants except CYP2C9*3 are rare in Han Chinese, with allele frequencies ranging from 0.03 to 0.18%. The total frequency of all CYP2C9*non-3 alleles is approximately 1%, which is lower than that reported by Dai et al. (10) and similar to the results of two studies on the Japanese population (24, 25). Considering the Chinese Han population size of approximately 1.3 billion and their functional significance, exploiting the distribution characteristics of rare *CYP2C9* allele variants has important clinical value for guiding the precise and individualized warfarin administration in Chinese Han patients despite low allele frequencies.

The warfarin maintenance dose is influenced by a variety of genetic and non-genetic factors. Many studies have shown that genetic factors mainly include *CYP2C9* (previous studies focused on *2 and *3); *VKORC1* and *CYP4F2* can explain 30–50% of the interindividual variability in warfarin dos requirements (16, 26, 27). Multiple clinical factors and demographic data (such as age, sex, race, weight, and concomitant medication) may influence the warfarin maintenance dose and may account for 10%–20% of warfarin dose variation. Additionally, approximately 40% of individual warfarin dose variations cannot be explained by existing research results, in which rare *CYP2C9* variants other than *2 and *3 may play a role. The 2017 CPIC guidelines also recommended considering the effects of rare variants, CYP2C9*5, *6, *8, and *11, when adjusting warfarin dosage in people of African ancestry (15). However, there is little evidence on how to adjust the warfarin dose for other rare variants, and guidelines do not specifically recommend it.

Our study systematically analyzed the effects of a series of rare CYP2C9*non-3 variants on stable warfarin doses in Han Chinese patients. The individual warfarin doses differed significantly in this study, with an 18-fold difference. This study reported the warfarin dose requirements in carriers of various rare *CYP2C9* variants. Among them, CYP2C9*1/*13, *1/*16, *1/*58, *1/*59, *1/*60, and *1/*62 were previously identified in patients with lower warfarin dose requirements (28–30). The warfarin dose requirements in carriers of CYP2C9*1/*27, *1/*29, *1/*31, *1/*33, *1/*39, *1/*42, and *1/*50 were reported for the first time. The mean warfarin dose in carriers of CYP2C9*non-3 significantly decreased. Many *in vivo* and *in vitro* studies have demonstrated that rare variants are often associated with decreased drug metabolic activity, and only a few rare variants show increased or unchanged metabolic activity (12, 14, 31). Two studies assessed the *in vitro* catalytic activities of over 30 rare alleles in the Chinese Han population. Of these variants, 85% showed reduced catalytic activity toward tolbutamide compared with the wild-type (12), and 94% displayed lower catalytic activities toward losartan (13). Rare *CYP2C9* variants can lead to spatial configuration instability of the enzyme and reduce substrate affinity (32). This is consistent with the finding that most carriers of rare *CYP2C9* variants had lower warfarin dose requirements in this study. CYP2C9*13 has the highest frequency (0.19%) in this study among the CYP2C9*non-3 variants and the warfarin maintenance dose in carriers with CYP2C9*1/*13 was significantly reduced to only 1.60 (± 0.28) mg/d. CYP2C9*13 has been widely reported in East Asian populations, with allele frequencies ranging from 0.05–0.61% (33–35). Studies *in vivo* on Chinese Han subjects found that CYP2C9*13 was associated with poor lornoxicam and losartan metabolism (33, 36). Carriers with CYP2C9*3/*13 dual variants have also been shown to have an extremely low stable warfarin dose (37). Our findings confirmed that, besides CYP2C9*3, CYP2C9*13 may be another noteworthy variant in the Chinese Han population.

The mean doses in individuals with CYP2C9*non-3 variants were significantly lower than those of wild-type carriers and comparable to those of CYP2C9*3. From the perspective of pharmacogenetics, the lower *VKORC1-1639G* allele frequency in the Chinese Han population may be an important reason why the warfarin maintenance dose in the Han Chinese is lower than that in patients of European and African descent (38). Moreover, carrying the CYP2C9*non-3 variant would further reduce the individual warfarin dose requirements. The definition of warfarin sensitivity in previous studies mainly refers to the genotype combination of *CYP2C9* (*2 and *3) and *VKORC1* (*-1639G* > *A*) from the USA FDA drug label (6). Due to the conspicuous differences in the pharmacogenetic background, this definition is not applicable to the Chinese Han population and cannot reflect the significance of CYP2C9*non- 3 variants. Therefore, in this study, we stratified the patients according to their actual warfarin doses and defined patients whose stable doses were within the lowest 20 and 10% range as warfarin-sensitive and highly sensitive responders, respectively. It is more direct and accurate, and administration of regular doses to these patients may lead to an increased bleeding risk. Moreover, SKAT-O analysis confirmed that CYP2C9*non-3 variants significantly correlated with warfarin sensitivity.

Previous studies have evaluated the effects of various genetic and non-genetic factors on the warfarin maintenance dose, mainly through multiple linear regression analysis (39). However, this method is only suitable for analyzing single-gene variants with higher frequencies. There are various rare *CYP2C9* variants in the Han Chinese population, with very low allele frequencies for single variants (10). Moreover, various variants have different effects on the metabolic activity of enzymes (12–14). Therefore, it is difficult to investigate the overall impact of rare variants using linear regression equations. In contrast, the SKAT analysis is an effective method for evaluating the cumulative effects of a group of rare variants. SKAT is a score-based variance component test that allows different variants to have different directions and magnitudes of effects and shows higher computational efficiency for various rare variants (40). SKAT-O was further refined based on the SKAT and Burden tests. It offers improved power and performs optimal tests (41). However, the SKAT analysis still has some limitations. The SKAT is a score test that only performs a hypothesis test, and the statistics cannot reflect the effect size of rare variants. This study suggested that the warfarin dose in CYP2C9*non-3 carriers was significantly lower than that of CYP2C9*1/*1. SKAT-O analysis confirmed that rare CYP2C9*non-3 variants are significantly associated with warfarin sensitivity. Similar to CYP2C9*3, the *non-3 variant has important implications for warfarin-sensitive and highly sensitive patients. Therefore, the role of CYP2C9*non-3 rare variants should not be ignored when exploring the genetic factors affecting stable warfarin doses in Han Chinese patients.

Furthermore, we found that individuals with lower warfarin dose requirements were more likely to carry CYP2C9*3 and *non-3 variants, and the proportion of CYP2C9*non-3 carriers increased with decreasing stable warfarin doses. At the same time, the proportions of warfarin-sensitive responders in CYP2C9*3 and *non-3 carriers were similar, and both were significantly higher than that of the wild-type. Compared with CYP2C9*3, *non-3 carriers had a higher proportion of highly sensitive responders. Therefore, CYP2C9*non-3 and *3 variants had similar values in screening warfarin-sensitive populations. CYP2C9*non-3 displayed a stronger predictive power for highly sensitive responders and should not be ignored. Our study confirmed for the first time that CYP2C9*non-3 has significant implications for warfarin sensitivity in Han Chinese individuals. The Engage AF-TIMI 48 study showed that the advantages of NOACs in reducing the bleeding risk compared with warfarin were mainly reflected in patients sensitive to warfarin (6). In our study, it can be inferred that screening for both CYP2C9*3 and CYP2C9*non-3 variants before anticoagulation therapy is reasonable in Han Chinese, since most (approximately 60%) variant carriers (regardless of CYP2C9*3 or *non-3 variants) are patients sensitive to warfarin, and optimized treatment with NOACs in this group may effectively reduce bleeding risk.

Previous studies on warfarin pharmacogenetics have focused on genotype-guided warfarin dose-prediction strategies (42–44). In this regard, the results of multiple randomized controlled trials (RCTs) were inconsistent. The EU-PACT and the GIFT studies demonstrated the clinical benefit of genotype-guided warfarin dosing (42, 43). However, the COAG study arrived at the opposite conclusion (44). Therefore, until now, there has been no clear recommendation on whether to use genotypes to guide warfarin dosing in clinical practice. Our study confirmed that CYP2C9*non-3 and CYP2C9*3 variants had similar significance, at least in the Chinese Han population, when screening warfarin-sensitive and highly sensitive patients. Identifying such patients using gene sequencing may improve warfarin therapy safety. Based on the available evidence, we proposed a new genotype-guided drug selection strategy, in which warfarin was administered to patients with normal warfarin-sensitive genotypes, and NOACs were preferred for carriers with CYP2C9*3 or CYP2C9*non-3 variants. The results of this study can help guide the formulation of individualized anticoagulant treatment regimens and drug selection for patients. Further prospective controlled clinical studies are required to evaluate the feasibility and effectiveness of this strategy in reducing the bleeding risk in patients receiving anticoagulation therapy.

This study had some limitations. First, as a retrospective study, it had the typical limitations of retrospective analyses. The conclusion remains to be validated by prospective studies. Second, the number of carriers of CYP2C9*non-3 variants in the study was small, and bias may have been present. Future studies are required to incorporate more carriers with rare alleles and ex plore the impact of rare *CYP2C9* alleles on warfarin doses in a larger sample. Third, the SKAT-O statistics only provided correlation analysis and did not reflect the effect size of rare variants. Fourth, more genetic factors, such as *CYP4F2* and *CYP2C19*, should be jointly used to predict individual warfarin sensitivity, considering the increasing clinical evidence for other genes (45, 46).

## Data availability statement

The raw data supporting the conclusions of this article will be made available by the authors, without undue reservation.

## Ethics statement

The studies involving human participants were reviewed and approved by the Ethics Committee of the Beijing Hospital. The patients/participants provided their written informed consent to participate in this study.

## Author contributions

DW, DD, and HC: study conception and design. DW, HW, and MD: analysis and interpretation of data, drafting of the article, and statistical expertise. QZ, AZ, XZ, JC, MHD, YW, HS, SW, FW, JPC, DD, and HC: critical revision of the article for intellectual content and final approval of the article. DW, HW, MD, QZ, AZ, XZ, JC, MHD, YW, HS, and SW: provision of study materials or patients. DW, QZ, AZ, XZ, JC, and MHD: administrative, technical, and logistic support. DW, QZ, AZ, and XZ: collection of data. All authors contributed to the article and approved the submitted version.

## References

[1] Ageno W Gallus AS Wittkowsky A Crowther M Hylek EM Palareti G. Oral anticoagulant therapy: antithrombotic therapy and prevention of thrombosis, 9th ed: American college of chest physicians evidence-based clinical practice guidelines. *Chest.* (2012) 141(Suppl. 2):e44S–e88S. 10.1378/chest.11-2292 22315269 PMC3278051

[2] van Gorp RH Schurgers LJ. New insights into the pros and cons of the clinical use of vitamin K antagonists (VKAs) versus direct oral anticoagulants (DOACs). *Nutrients.* (2015) 7:9538–57. 10.3390/nu7115479 26593943 PMC4663607

[3] Mekaj YH Mekaj AY Duci SB Miftari EI. New oral anticoagulants: their advantages and disadvantages compared with vitamin K antagonists in the prevention and treatment of patients with thromboembolic events. *Ther Clin Risk Manag.* (2015) 11:967–77. 10.2147/tcrm.S84210 26150723 PMC4485791

[4] Gomes T Mamdani MM Holbrook AM Paterson JM Hellings C Juurlink DN. Rates of hemorrhage during warfarin therapy for atrial fibrillation. *CMAJ.* (2013) 185:E121–7. 10.1503/cmaj.121218 23184840 PMC3563912

[5] Wadelius M Pirmohamed M. Pharmacogenetics of warfarin: current status and future challenges. *Pharmacogenomics J.* (2007) 7:99–111. 10.1038/sj.tpj.6500417 16983400

[6] Mega JL Walker JR Ruff CT Vandell AG Nordio F Deenadayalu N Genetics and the clinical response to warfarin and edoxaban: findings from the randomised, double-blind ENGAGE AF-TIMI 48 trial. *Lancet.* (2015) 385:2280–7. 10.1016/s0140-6736(14)61994-2 25769357

[7] Yang J Chen Y Li X Wei X Chen X Zhang L Influence of CYP2C9 and VKORC1 genotypes on the risk of hemorrhagic complications in warfarin-treated patients: a systematic review and meta-analysis. *Int J Cardiol.* (2013) 168:4234–43. 10.1016/j.ijcard.2013.07.151 23932037

[8] Kaye JB Schultz LE Steiner HE Kittles RA Cavallari LH Karnes JH. Warfarin pharmacogenomics in diverse populations. *Pharmacotherapy.* (2017) 37:1150–63. 10.1002/phar.1982 28672100 PMC6913521

[9] Zhang J Chen Z Chen C. Impact of CYP2C9, VKORC1 and CYP4F2 genetic polymorphisms on maintenance warfarin dosage in Han-Chinese patients: a systematic review and meta-analysis. *Meta Gene.* (2016) 9:197–209. 10.1016/j.mgene.2016.07.002 27617219 PMC5006145

[10] Dai DP Xu RA Hu LM Wang SH Geng PW Yang JF CYP2C9 polymorphism analysis in Han Chinese populations: building the largest allele frequency database. *Pharmacogenomics J.* (2014) 14:85–92. 10.1038/tpj.2013.2 23400009

[11] Ji Y Chen S Zhao L Pan P Wang L Cai J In vitro assessment of 39 CYP2C9 variants found in the Chinese population on the metabolism of the model substrate fluoxetine and a summary of their effects on other substrates. *J Clin Pharm Ther.* (2015) 40:320–7. 10.1111/jcpt.12267 25884291

[12] Dai DP Wang YH Wang SH Geng PW Hu LM Hu GX In vitro functional characterization of 37 CYP2C9 allelic isoforms found in Chinese Han population. *Acta Pharmacol Sin.* (2013) 34:1449–56. 10.1038/aps.2013.123 24077631 PMC4006465

[13] Wang YH Pan PP Dai DP Wang SH Geng PW Cai JP Effect of 36 CYP2C9 variants found in the Chinese population on losartan metabolism in vitro. *Xenobiotica.* (2014) 44:270–5. 10.3109/00498254.2013.820007 23844998

[14] Hu GX Pan PP Wang ZS Yang LP Dai DP Wang SH In vitro and in vivo characterization of 13 CYP2C9 allelic variants found in Chinese Han population. *Drug Metab Dispos.* (2015) 43:561–9. 10.1124/dmd.114.061200 25614704

[15] Johnson JA Caudle KE Gong L Whirl-Carrillo M Stein CM Scott SA Clinical pharmacogenetics implementation consortium (CPIC) guideline for pharmacogenetics-guided warfarin dosing: 2017 update. *Clin Pharmacol Ther.* (2017) 102:397–404. 10.1002/cpt.668 28198005 PMC5546947

[16] Klein TE Altman RB Eriksson N Gage BF Kimmel SE Lee MT Estimation of the warfarin dose with clinical and pharmacogenetic data. *N Engl J Med.* (2009) 360:753–64. 10.1056/NEJMoa0809329 19228618 PMC2722908

[17] Johnson JA Cavallari LH. Warfarin pharmacogenetics. *Trends Cardiovasc Med.* (2015) 25:33–41. 10.1016/j.tcm.2014.09.001 25282448 PMC4278947

[18] Cavallari LH Langaee TY Momary KM Shapiro NL Nutescu EA Coty WA Genetic and clinical predictors of warfarin dose requirements in African Americans. *Clin Pharmacol Ther.* (2010) 87:459–64. 10.1038/clpt.2009.223 20072124

[19] Limdi NA Arnett DK Goldstein JA Beasley TM McGwin G Adler BK Influence of CYP2C9 and VKORC1 on warfarin dose, anticoagulation attainment and maintenance among European-Americans and African-Americans. *Pharmacogenomics.* (2008) 9:511–26. 10.2217/14622416.9.5.511 18466099 PMC2757655

[20] Lindley KJ Limdi NA Cavallari LH Perera MA Lenzini P Johnson JA Warfarin dosing in patients with CYP2C9*5 variant alleles. *Clin Pharmacol Ther.* (2022) 111:950–5. 10.1002/cpt.2549 35108398 PMC13108523

[21] O’Brien TJ Kidd RS Richard CA Ha NH Witcher P Tran LV First report of warfarin dose requirements in patients possessing the CYP2C9*12 allele. *Clin Chim Acta.* (2013) 424:73–5. 10.1016/j.cca.2013.05.008 23688605

[22] Lee MY Borgiani P Johansson I Oteri F Mkrtchian S Falconi M High warfarin sensitivity in carriers of CYP2C9*35 is determined by the impaired interaction with P450 oxidoreductase. *Pharmacogenomics J.* (2014) 14:343–9. 10.1038/tpj.2013.41 24322786

[23] Ciccacci C Falconi M Paolillo N Oteri F Forte V Novelli G Characterization of a novel CYP2C9 gene mutation and structural bioinformatic protein analysis in a warfarin hypersensitive patient. *Pharmacogenet Genomics.* (2011) 21:344–6. 10.1097/FPC.0b013e328344c340 21451434

[24] Yin T Maekawa K Kamide K Saito Y Hanada H Miyashita K Genetic variations of CYP2C9 in 724 Japanese individuals and their impact on the antihypertensive effects of losartan. *Hypertens Res.* (2008) 31:1549–57. 10.1291/hypres.31.1549 18971529

[25] Maekawa K Fukushima-Uesaka H Tohkin M Hasegawa R Kajio H Kuzuya N Four novel defective alleles and comprehensive haplotype analysis of CYP2C9 in Japanese. *Pharmacogenet Genomics.* (2006) 16:497–514. 10.1097/01.fpc.0000215069.14095.c6 16788382

[26] Baker WL Johnson SG. Pharmacogenetics and oral antithrombotic drugs. *Curr Opin Pharmacol.* (2016) 27:38–42. 10.1016/j.coph.2016.01.008 26878737

[27] Perini JA Struchiner CJ Silva-Assuncao E Santana IS Rangel F Ojopi EB Pharmacogenetics of warfarin: development of a dosing algorithm for brazilian patients. *Clin Pharmacol Ther.* (2008) 84:722–8. 10.1038/clpt.2008.166 18754001

[28] Wang D Dai DP Wu H Chong J Lü Y Yin R Effects of rare CYP2C9 alleles on stable warfarin doses in Chinese Han patients with atrial fibrillation. *Pharmacogenomics.* (2020) 21:1021–31. 10.2217/pgs-2020-0051 32893731

[29] Dai DP Wang SH Li CB Geng PW Cai J Wang H Identification and functional assessment of a new CYP2C9 allelic variant CYP2C9*59. *Drug Metab Dispos.* (2015) 43:1246–9. 10.1124/dmd.115.063412 25994031

[30] Chen H Dai DP Zhou S Liu J Wang SH Wu HL An identification and functional evaluation of a novel CYP2C9 variant CYP2C9*62. *Chem Biol Interact.* (2020) 327:109168. 10.1016/j.cbi.2020.109168 32531309

[31] Niinuma Y Saito T Takahashi M Tsukada C Ito M Hirasawa N Functional characterization of 32 CYP2C9 allelic variants. *Pharmacogenomics J.* (2014) 14:107–14. 10.1038/tpj.2013.22 23752738

[32] Kumondai M Ito A Gutiérrez Rico EM Hishinuma E Ueda A Saito S Functional assessment of 12 rare allelic CYP2C9 variants identified in a population of 4773 Japanese individuals. *J Pers Med.* (2021) 11:94. 10.3390/jpm11020094 33540768 PMC7912942

[33] Li Z Wang G Wang LS Zhang W Tan ZR Fan L Effects of the CYP2C9*13 allele on the pharmacokinetics of losartan in healthy male subjects. *Xenobiotica.* (2009) 39:788–93. 10.1080/00498250903134435 19604036

[34] Bae JW Choi CI Kim MJ Oh DH Keum SK Park JI Frequency of CYP2C9 alleles in Koreans and their effects on losartan pharmacokinetics. *Acta Pharmacol Sin.* (2011) 32:1303–8. 10.1038/aps.2011.100 21841812 PMC4010224

[35] Lee HW Lim MS Lee J Jegal MY Kim DW Lee WK Frequency of CYP2C9 variant alleles, including CYP2C9*13 in a Korean population and effect on glimepiride pharmacokinetics. *J Clin Pharm Ther.* (2012) 37:105–11. 10.1111/j.1365-2710.2010.01238.x 21208246

[36] Si D Guo Y Zhang Y Yang L Zhou H Zhong D. Identification of a novel variant CYP2C9 allele in Chinese. *Pharmacogenetics.* (2004) 14:465–9. 10.1097/01.fpc.0000114749.08559.e4 15226678

[37] Kwon MJ On YK Huh W Ko JW Kim DK Kim JS Low dose requirement for warfarin treatment in a patient with CYP2C9*3/*13 genotype. *Clin Chim Acta.* (2011) 412:2343–5. 10.1016/j.cca.2011.06.040 21782804

[38] Limdi NA Wadelius M Cavallari L Eriksson N Crawford DC Lee MT Warfarin pharmacogenetics: a single VKORC1 polymorphism is predictive of dose across 3 racial groups. *Blood.* (2010) 115:3827–34. 10.1182/blood-2009-12-255992 20203262 PMC2865873

[39] Asiimwe IG Zhang EJ Osanlou R Jorgensen AL Pirmohamed M. Warfarin dosing algorithms: a systematic review. *Br J Clin Pharmacol.* (2020) 87:1717–29. 10.1111/bcp.14608 33080066 PMC8056736

[40] Wu MC Lee S Cai T Li Y Boehnke M Lin X. Rare-variant association testing for sequencing data with the sequence kernel association test. *Am J Hum Genet.* (2011) 89:82–93. 10.1016/j.ajhg.2011.05.029 21737059 PMC3135811

[41] Lee S Wu MC Lin X. Optimal tests for rare variant effects in sequencing association studies. *Biostatistics.* (2012) 13:762–75. 10.1093/biostatistics/kxs014 22699862 PMC3440237

[42] Pirmohamed M Burnside G Eriksson N Jorgensen AL Toh CH Nicholson T A randomized trial of genotype-guided dosing of warfarin. *N Engl J Med.* (2013) 369:2294–303. 10.1056/NEJMoa1311386 24251363

[43] Gage BF Bass AR Lin H Woller SC Stevens SM Al-Hammadi N Effect of genotype-guided warfarin dosing on clinical events and anticoagulation control among patients undergoing hip or knee arthroplasty: the GIFT randomized clinical trial. *JAMA.* (2017) 318:1115–24. 10.1001/jama.2017.11469 28973620 PMC5818817

[44] Kimmel SE French B Kasner SE Johnson JA Anderson JL Gage BF A pharmacogenetic versus a clinical algorithm for warfarin dosing. *N Engl J Med.* (2013) 369:2283–93. 10.1056/NEJMoa1310669 24251361 PMC3942158

[45] Liang R Wang C Zhao H Huang J Hu D Sun Y. Influence of CYP4F2 genotype on warfarin dose requirement-a systematic review and meta-analysis. *Thromb Res.* (2012) 130:38–44. 10.1016/j.thromres.2011.11.043 22192158

[46] Wang D Yong L Zhang Q Chen H. Impact of CYP2C19 gene polymorphisms on warfarin dose requirement: a systematic review and meta-analysis. *Pharmacogenomics.* (2022) 23:903–11. 10.2217/pgs-2022-0106 36222113

