# Supplementary material for: Clinical significance of the series of CYP2C9*non3 variants, an unignorable predictor of warfarin sensitivity in Chinese population
Source: Front Cardiovasc Med. 2022 Nov 24;9:1052521. doi: 10.3389/fcvm.2022.1052521 (PMC9729276; doi:10.3389/fcvm.2022.1052521)
Supplement: Supplementary file 1 [file Image_1.pdf]

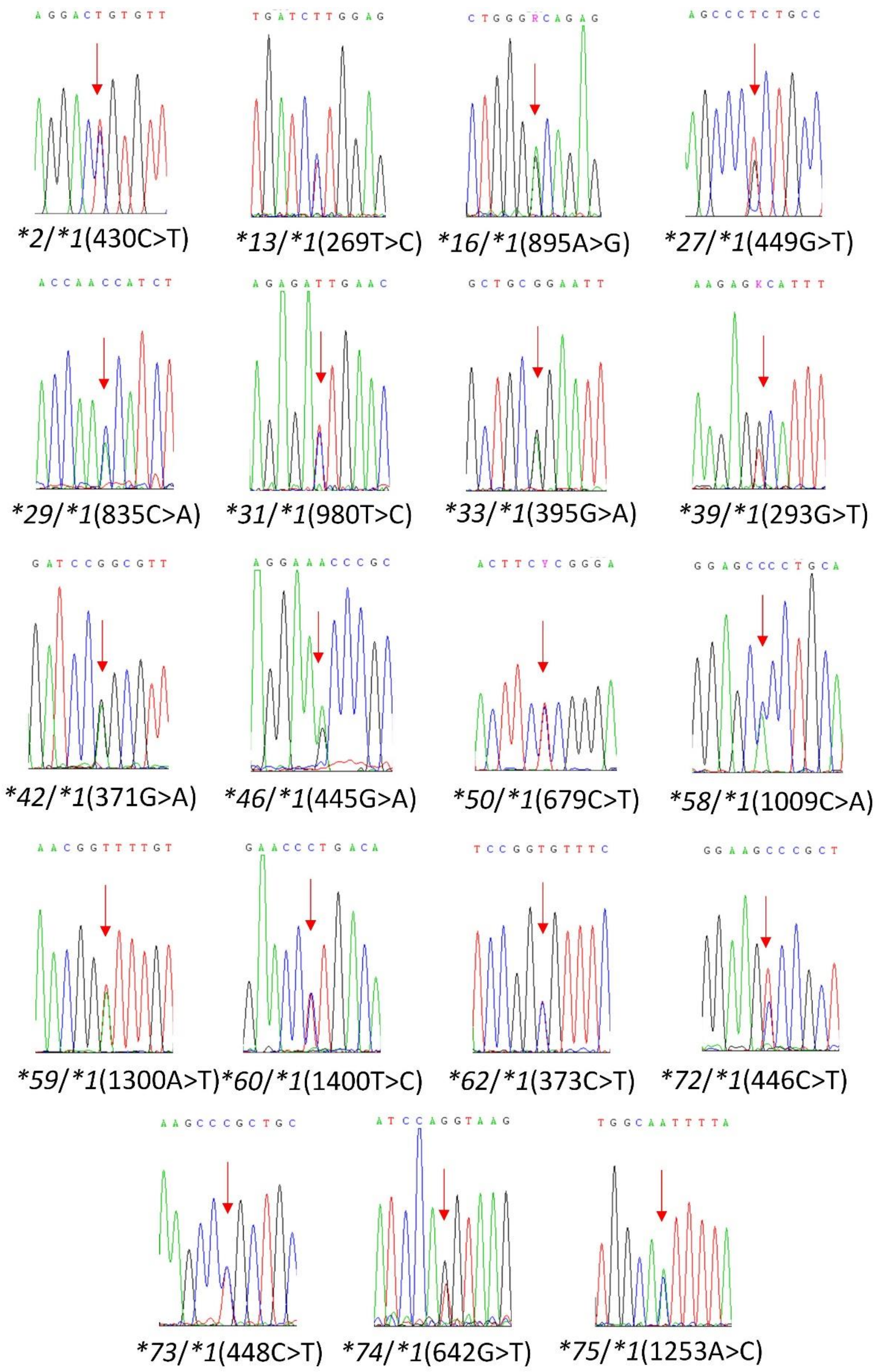

Supplemental Figure: Sequencing captures of individuals carrying *non-3 allelic variants of *CYP2C9*

Genotypes and cDNA mutated sites are noted and red arrows refer to the mutated sites in carriers.
